# On Guard in Wartime

**Published:** 1917-02-10

**Authors:** 


### ON GUARD IN WARTIME.

Germany has notified the world that her practice from the present time onwards will be for her submarines to fire upon and sink without warning all ships, including neutrals, and even hospital ships. The enemy are full of threats, all aimed at the destruction of England and everything English. The treatment of America by the Germans has given us as a nation pause to think, and there has been evidence since Germany's declaration of this policy of unmeasured barbarism that hospitals—and, we may take it, any institution or place throughout these islands, where there are possible means of distributing, or obtaining, aids to promote death or injury, are liable to be utilised by our enemies at any time. Poison has already been resorted to by the Huns in the course of this war. There has been evidence of inoculations of dangerous and fatal compounds. We must anticipate the use of poisons in any form, where devilish ingenuity can purchase or snatch an opportunity for their use. All these ills should be provided against to the utmost.

Hospitals and dispensaries for the treatment of the sick everywhere throughout the country, and all laboratories in connection with universities and elsewhere, are centres which demand the overhauling of the existing arrangements in regard to the custody and safe keeping of poisons. As recently as this week striking evidence has been given that demonstrates this necessity. In this connection we may point out that it is the practice in many hospitals to keep the poisons in a locked cupboard, which is frequently to be found in the ward, but sometimes in the clinical or sister's room, or in the ward kitchen on each floor. In the course of our inspections of hospitals of all types our experience has taught us that, in more than 50 per cent. of these cupboards the inspector with a keen eye may find the key in the door. Familiarity breeds contempt, and the habitual custody of dangerous remedies and their use, may lead to a loss of apprehension of the risks and dangers attending carelessness in relation to the custody of dangerous drugs.

The present war conditions should enforce the imperative necessity for the readjustment and rearrangement, not only of the custody but of the distribution of poisons of all kinds everywhere throughout the country. We are confident that we have only to call attention to this matter to secure that the authorities immediately concerned will take the necessary steps to close the door effectually, against any Hunnish attempts to further their vile ends by seizing upon any weak points there may happen to be in the machinery and arrangements for the custody and security of poisons in hospitals, institutions, and elsewhere throughout the country. It is a golden rule to put all poisons in the custody of responsible officers who are held personally liable for their use and abuse.

